# The Potential for Enhancing the Power of Genetic Association Studies in African Americans through the Reuse of Existing Genotype Data

**DOI:** 10.1371/journal.pgen.1001096

**Published:** 2010-09-02

**Authors:** Gary K. Chen, Robert C. Millikan, Esther M. John, Christine B. Ambrosone, Leslie Bernstein, Wei Zheng, Jennifer J. Hu, Stephen J. Chanock, Regina G. Ziegler, Elisa V. Bandera, Brian E. Henderson, Christopher A. Haiman, Daniel O. Stram

**Affiliations:** 1 Department of Preventive Medicine, Keck School of Medicine, University of Southern California/Norris Comprehensive Cancer Center, Los Angeles, California, United States of America; 2 Department of Epidemiology, Gillings School of Global Public Health, and Lineberger Comprehensive Cancer Center, University of North Carolina, Chapel Hill, North Carolina, United States of America; 3 Northern California Cancer Center, Fremont, California, United States of America; 4 Stanford University School of Medicine and Stanford Cancer Center, Stanford, California, United States of America; 5 Department of Cancer Prevention and Control, Roswell Park Cancer Institute, Buffalo, New York, United States of America; 6 Division of Cancer Etiology, Department of Population Science, Beckman Research Institute, City of Hope, California, United States of America; 7 Division of Epidemiology, Department of Medicine, Vanderbilt Epidemiology Center, Nashville, Tennessee, United States of America; 8 Vanderbilt-Ingram Cancer Center, Vanderbilt University School of Medicine, Nashville, Tennessee, United States of America; 9 Sylvester Comprehensive Cancer Center and Department of Epidemiology and Public Health, University of Miami Miller School of Medicine, Miami, Florida, United States of America; 10 Epidemiology and Biostatistics Program, Division of Cancer Epidemiology and Genetics, National Cancer Institute, Bethesda, Maryland, United States of America; 11 The Cancer Institute of New Jersey, Robert Wood Johnson Medical School, New Brunswick, New Jersey, United States of America; Queensland Institute of Medical Research, Australia

## Abstract

We consider the feasibility of reusing existing control data obtained in genetic association studies in order to reduce costs for new studies. We discuss controlling for the population differences between cases and controls that are implicit in studies utilizing external control data. We give theoretical calculations of the statistical power of a test due to Bourgain et al (Am J Human Genet 2003), applied to the problem of dealing with case-control differences in genetic ancestry related to population isolation or population admixture. Theoretical results show that there may exist bounds for the non-centrality parameter for a test of association that places limits on study power even if sample sizes can grow arbitrarily large. We apply this method to data from a multi-center, geographically-diverse, genome-wide association study of breast cancer in African-American women. Our analysis of these data shows that admixture proportions differ by center with the average fraction of European admixture ranging from approximately 20% for participants from study sites in the Eastern United States to 25% for participants from West Coast sites. However, these differences in average admixture fraction between sites are largely counterbalanced by considerable diversity in individual admixture proportion within each study site. Our results suggest that statistical correction for admixture differences is feasible for future studies of African-Americans, utilizing the existing controls from the African-American Breast Cancer study, even if case ascertainment for the future studies is not balanced over the same centers or regions that supplied the controls for the current study.

## Author Summary

This paper discusses and provides unique insight into an important problem raised by the current state of genetic studies into disease susceptibility, namely whether we can reuse genetic data from participants genotyped as controls in one study when cases (people with a disease of interest) are obtained from other studies, or whether each new study needs its own controls. We are interested in whether studies where cases and controls are sampled differently will give correct answers and are as powerful statistically as when new control data is also genotyped. Because of the huge investments made recently in large scale genotyping of cases and controls for various diseases, this is a timely question. This question is especially important in understanding the genetic causes of disease in as-yet relatively understudied population groups, such as African-Americans, in order to speed up progress when this is possible. We give theoretical results about the power of studies that reuse existing control genotypes based on statistical considerations. We also provide analysis of real data from a major study of the genetic causes of breast cancer in African-American women in order to shed practical light upon this issue.

## Introduction

A genetic association study estimating the main effects of single nucleotide polymorphisms (SNPs) or other genetic variants upon the risk of a rare or common disease in minority populations is a setting in which it is especially attractive to consider the use of existing genotype data as a supplementary or even a primary source of controls. DNA samples may be expensive and difficult to obtain, and response rates are often lower in minority populations [1]. Researchers might consider using an already available “stand in” population sample as controls, provided that genotype frequencies are equivalent to those in the population from which controls would be drawn. There are however two immediate concerns raised, one fundamental and the other technical in nature: The fundamental question is whether or not the controls are sampled from the same underlying population (or populations) as are the cases – or more generally the feasibility and “cost” (generally loss of statistical power) of controlling for case/control differences if they arise. The technical question is whether differences in genotyping, including differences in DNA preparation, and in the actual markers genotyped in cases and controls, i.e. when the platforms are not identical so that imputation is relied upon to make up the difference, may introduce false positive (or false negative) associations.

Consider the problem of conducting a genetic association study aimed at discovering genetic variants related to the risk of a disease, where there already exists extensive genotyping data, perhaps publicly available, for members of similar populations. If the disease under consideration is rare (so that genotype frequencies for controls may be expected to be the same as in the general population) then it is intuitively appealing to consider using existing control data from studies of other rare diseases (or population-based studies if they exist) to reduce the cost or increase the statistical power of an association study. From the classical case-control literature, a study that uses 1∶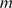 matching of controls to each of 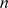 cases will be equivalent in power to a 1∶1 matched study with 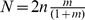 cases (and an equal number of controls). Thus a study with a large number of controls, 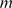, for each case will have nearly twice the effective sample size of a 1∶1 matched study [2].

Note that 1∶m matched studies (with m>1) are only cost effective if it is more costly or difficult to obtain additional cases than it is to obtain additional suitable controls since the increment in effective sample by (for example) doubling the number of case-control pairs (in a 1∶1 matching) is twice that of adding two new controls to each existing pair (to achieve 1∶3 matching) to a study even though the total number of participants is the same. If however the cost of adding an additional control is far less than that of adding an additional case (because control data is already available) then adding the almost “free” controls is highly attractive although the returns diminish as more and more controls are added, with the increment in effective sample size governed by m/(m+1).

This paper considers these issues from both theoretical and empirical perspectives. We apply a recent generalization [3]–[6] of the testing procedure of Bourgain et al [7] to the situation where population substructure (in the broad sense), including admixture and relatedness between subjects, is estimated from marker data rather than being assumed to be known as the basis for our theoretical considerations of study power. We point out below the relationship between this procedure and that of the more widely known principal components technique [8]. Our empirical investigation of the use of existing controls utilizes data from a genome-wide association study (GWAS) of breast cancer in African American women, namely the African American Breast Cancer (AABC) study, in which cases and controls come from a total of 9 different studies widely distributed geographically throughout the United States. African Americans are a relatively understudied group (compared to European Americans) in studies of genetic susceptibility. African Americans are admixed with Europeans and (in some cases) with Hispanics (themselves an admixed group) and Native Americans [9], [10]. We examine empirically the false positive rates that occur when cases from one geographical location or study within the AABC study are combined with controls from other AABC locations or studies, as well as the success (and cost in terms of loss of effective sample size) of adjustment for the observed population differences in global genetic ancestry when analyzing such illustrative data sets derived from the AABC study.

## Methods

### Statistical Methods

We utilize an approach derived from that of Bourgain et al [7] which has been discussed extensively in recent papers [3]–[5]. This approach for accounting for relatedness between subjects in association tests adopts a “retrospective” approach towards the problem of testing for disease associations using marker data, in which (as in the Armitage test) the allele frequency of a variant is related to case-control status. In vector notation we have 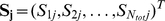 of observed values for a given SNP *j*. Here 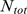 is the total number of subjects (cases+controls) in the study, and SNP values 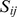 are coded as (0,1,2) for the number of copies of a specified allele, 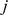, carried by subject 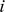. (This coding of SNP genotype implies that we are interested in additive models for the relationship between disease risk and genotype but the approach can readily be extended to other codings). The retrospective approach models the mean of 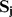 as a function of case-control status. If we define the 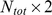 design matrix **C** to have rows (1 *c_i_*) where *c_i_* is case-control status (0 or 1) for subject *i* then the mean, 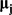, of 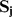 is written as
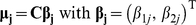
Relatedness between subjects induces a covariance matrix for the number of copies of a given SNP of form
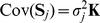
(1)with 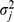 specific to each SNP but with the same matrix 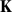 for all SNPs. In fact, for known pedigree relationships, and unrelated founders, this matrix 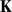 has diagonal elements equal to 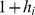 where 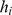 is the inbreeding coefficient for subject 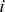 and each off-diagonal element, 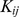, is twice the kinship coefficient for the relationship between subjects 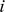 and 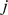 [7].

It is worth noting that in general the topic of relatedness includes what is often considered to be population substructure. For example consider two large but isolated populations (freely mixing within each population) that have been separated for many generations. While a random sample of people from the same population (with sample size small relative to the population size) might be considered unrelated to each other when considering that population separately, when considering the two populations together people from one isolated population are considered to be related to each other, relative to those in the other population. In particular, genetic markers will, through a process of random drift and other factors, be able to distinguish members from the two populations, and this will be detectable when calculation of the 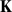 matrix is performed. A standard method of simulating genetic markers for divergent populations stemming from the same ancestral population (e.g. the Balding-Nichols model [11]) can readily be shown to produce covariance matrices of the form of expression (1).

If 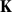 and 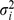 are both known then the best linear unbiased estimate (BLUE) of the regression vector 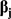 is of weighted least squares form
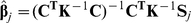
and the variance covariance matrix of the estimates is in the form of
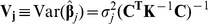
Thus inference on the significance of the allele frequency difference between cases and controls may be based upon the Wald test statistic
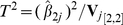
(2)with 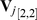 the (2,2) element of 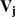


In general of course, 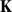 and 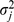 are not known, except in the case of known pedigrees and unrelated founders, where 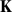 can be computed from first principles. The estimation of 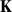 using marker data has been considered by a number of authors and both method of moments [4], [5] and maximum likelihood methods [3] have been considered. A method of moments estimator of 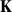 can be concisely written [5] as
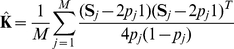
(3)and the estimate of 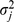 as
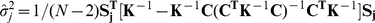
One value of this approach, which is exploited here, is that it is relatively easy to compute the power of the Wald 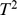 test if we can hypothesize a form of the relatedness matrix 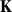. For a given form for 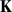 (below we consider several forms for both isolated population models and more complex admixed populations) then for a given sample size, 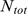, a given allele frequency for a causal SNP, and a hypothesized difference in allele frequencies between cases and controls (which can then be related to odds ratios in typical case/control analysis) we can compute the non-centrality parameter of 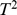 (and hence the power of the test) as
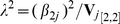
(4)We illustrate the computation of this non-centrality parameter for a number of important special cases in the results section below. It is worth noting now, however, that the Bourgain test appears to be reasonably powerful compared to other procedures, and can sometimes be considered as a compromise between the principal components method [8] and genomic control [12]. We attempt to justify this last statement in the results section below.

In addition to the Bourgain test we used several well known tools for addressing population structure in the AABC data. For example we computed eigenvectors through the use of the program EIGENSTRAT [8]. Briefly, each eigenvector explains a proportion of the genetic variation among samples in the analysis so that the leading eigenvector explains the greatest variation, followed by the second eigenvector, and so forth. The full set of eigenvectors form an orthonormal basis so that each eigenvector is scaled on the unit interval and linearly independent from all other eigenvectors. Note that the EIGENSTRAT procedure is operating on the same estimated 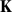 matrix, 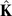, that we have described above.

To assess ancestry within the AABC study in relation to reference populations from HapMap, we performed a principal components analysis based on ancestry informative markers that were genotyped in both the AABC study and the HapMap Phase 3 populations. The 2,546 ancestry informative markers (contained within the Illumina 1M genotyping array which was used in the AABC scan) were selected based on low inter-marker correlation and high correlation to a previously determined eigenvector that explained African and European ancestry.

We quantified percent African ancestry for each of the nine AABC study populations by running the program STRUCTURE for each study population. The program implements a Markov Chain Monte Carlo algorithm that provides the posterior estimates of the proportion of ancestry from each of k clusters for each individual, where k is specified by the investigator. For each AABC study population, we assigned k = 3, including genotypes from the same ancestry informative markers used in PCA genotyped in YRI, CEU, and JPT from HapMap Phase 3.

### Studies in AABC

AABC included 9 epidemiological studies of breast cancer among African American women, which comprise a total of 3,153 cases and 2,831 controls. Below is a brief description of these studies.

#### The Multiethnic Cohort Study (MEC)

The MEC is a prospective cohort study of 215,000 men and women in Hawaii and Los Angeles [1] between the ages of 45 and 75 years at baseline (1993–1996). Through December, 31 2007, a nested breast cancer case-control study in the MEC included 556 African American cases (554 invasive and 12 in situ) and 1,003 African American controls. An additional 178 African American breast cancer cases (ages: 50–84) diagnosed between June 1, 2006 and December 31, 2007 in Los Angeles County (but outside of the MEC) were combined with the MEC samples in the analysis.

#### The Los Angeles component of The Women's Contraceptive and Reproductive Experiences (CARE) Study

The NICHD Women's CARE Study is a large multi-center population-based case-control study that was designed to examine the effects of oral contraceptive (OC) use on invasive breast cancer risk among African American women and white women ages 35–64 years in five U.S. locations [13]. Cases in Los Angeles County were diagnosed from July 1, 1994 through April 30, 1998, and controls were sampled by random-digit dialing (RDD) from the same population and time period; 380 African American cases and 224 African American controls were included in stage 1 of the scan.

#### The Women's Circle of Health Study (WCHS)

The WCHS is an ongoing case-control study of breast cancer among women of European and African descent residing in the New York City boroughs (Manhattan, the Bronx, Brooklyn and Queens) and in seven counties in New Jersey (Bergen, Essex, Hudson, Mercer, Middlesex, Passaic, and Union) [14]. Eligible cases included women diagnosed with invasive breast cancer between 20 and 74 years of age; controls were identified through RDD. The WCHS contributed 272 invasive African American cases and 240 African American controls to stage 1 of the GWAS.

#### The San Francisco Bay Area Breast Cancer Study (SFBC)

The SFBC is a population-based case-control study of invasive breast cancer in Hispanic, African American and non-Hispanic White women conducted between 1995 and 2003 in the San Francisco Bay Area [15]. African American cases, ages 35–79 years, were diagnosed between April 1, 1995 and April 30, 1999, with controls identified through RDD. Stage 1 included 172 invasive African American cases and 231 African American controls from SFBC.

#### The Northern California Breast Cancer Family Registry (NC–BCFR)

The NC-BCFR is an on-going population-based family study conducted in the Greater San Francisco Bay Area, and is one of 6 sites collaborating in the Breast Cancer Family Registry (BCFR), an international consortium funded by NCI [16]. African American breast cancer cases in NC-BCFR were diagnosed after January 1, 1995 and between the ages of 18 and 64 years; population controls were identified through random digit dialing (RDD). Stage 1 genotyping was conducted for 440 invasive African American cases and 53 African American controls.

#### The Carolina Breast Cancer Study (CBCS)

The CBCS is a population-based case-control study conducted between 1993 and 2001 in 24 counties of central and eastern North Carolina [17]. Cases were identified by rapid case ascertainment system in cooperation with the North Carolina Central Cancer Registry and controls were selected from the North Carolina Division of Motor Vehicle and United States Health Care Financing Administration beneficiary lists. Participants' ages ranged from 20 to 74 years. For stage 1, DNA samples were provided from 656 African American cases with invasive breast cancer and 608 African American controls.

#### The Prostate, Lung, Colorectal, and Ovarian Cancer Screening Trial (PLCO) Cohort

PLCO, coordinated by the U.S. National Cancer Institute (NCI) in 10 U.S. centers, enrolled during 1993–2001 approximately 155,000 men and women, aged 55–74 years, in a randomized, two-arm trial to evaluate the efficacy of screening for these four cancers [18]. A total of 64 African American invasive breast cancer cases and 133 African American controls contributed to stage 1 of the GWAS.

#### The Nashville Breast Health Study (NBHS)

The NBHS is a population-based case-control study of incident breast cancer conducted in the Tennessee [19]. The study was initiated in 2001 to recruit patients with invasive breast cancer or ductal carcinoma *in situ*, and controls, recruited through RDD between the ages of 25 and 75 years. NBHS contributed 310 African American invasive cases (57 in situ), and 186 African American controls to the stage 1 analysis.

#### Wake Forest University Breast Cancer Study (WFBC)

African American breast cancer cases and controls in WFBC were recruited at Wake Forest University Health Sciences from November 1998 through December 2008 [20]. Controls were recruited from the patient population receiving routine mammography at the Breast Screening and Diagnostic Center. Age range of participants was 30–86 years. WFBC contributed 125 cases (116 invasive and 9 in situ) and 153 controls to the stage 1 analysis.

### Genotyping

Genotyping in stage 1 was conducted using the Illumina Human1M-Duo BeadChip. Of the 5,984 samples from these studies (3,153 cases and 2,831 controls), we attempted genotyping of 5,932, removing samples (n = 52) with DNA concentrations <20 ng/ul by pico green assay. After clustering the genotype data we removed samples based on the following exclusion criteria: 1) unknown replicates (≥98.9% genetically identical) that we were able to confirm (only one of each duplicate was removed, n = 15); 2) unknown replicates that we were not able to confirm through discussions with study investigators (pair or triplicate removed, n = 14); 3) samples with call rates <95% after a second attempt (n = 100); 4) samples with ≤5% African ancestry (n = 36) (discussed below); and, 5) samples with <15% mean heterozygosity of SNPs in the X chromosome and/or similar mean allele intensities of SNPs on the X and Y chromosomes (n = 6) (these are likely to be males).

In the analysis, we removed SNPs with <95% call rate or minor allele frequencies (MAFs) <1%. To assess genotyping reproducibility we included 138 replicate samples; the average concordance rate was 99.95% (>99.93% for all pairs). We also eliminated SNPs with genotyping concordance rates <98% based on the replicates. The final analysis dataset included 3,016 cases and 2,745 controls, with an average SNP call rate of 99.7% and average sample call rate of 99.8%. Hardy-Weinberg equilibrium (HWE) was not used as a criterion for removing SNPs for this analysis.

## Results

### Non-Centrality Parameter for the Bourgain Test in the Case of Isolated Populations

We use the Balding Nichols model [11] for allele frequency differences between isolated populations. In this model allele frequencies for a SNP in modern data populations are distributed according to a beta distribution 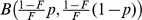 with 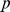 the ancestral allele frequency of that SNP. In this model the variance of the modern day allele frequency is 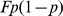, thus 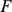 is a parameter specifying the degree of separation between the modern day and ancestral population. As described in Rakovski and Stram 2009 [4] if genotypes are obtained for randomly sampled individual from two modern day isolated populations using this model and the separation of each modern day population from the ancestral population equals 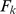 (for 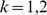) statistic then the covariance matrix, 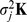 between subjects for the jth SNP will have diagonal terms equal to 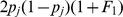 for members of the first population, diagonal terms equal to 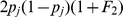 for members of the second, off diagonal terms of 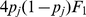, 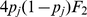, or zero for pairs of individuals who are either both from the first population, both from the second population or from different populations respectively. Here 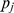 is the frequency in the ancestral population of SNP 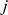. Consider now a study in which all cases come from one isolated population, and all controls from another. Assume for simplicity that 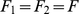 (both populations have the same degree of separation from their ancestral source), and that the number of cases and controls are both equal to 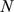 so that total sample size is 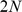 (the calculations below can be readily altered for different matching fractions if necessary). Thus we can write the variance of the estimator of the case-control difference for SNP 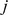 as
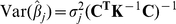
with the first column of **C** being a vector of 1's and the second column of **C** a vector of 1's and 0's indicating case-control (and population) status. Using a readily derived formula for the inverse of an 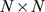 matrix of compound symmetric form
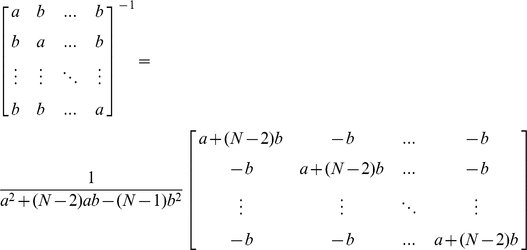
we can easily write
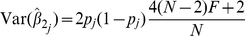
(5)Thus the non-centrality parameter, 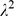 = 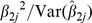 of a test of association does not increase linearly in N, but rather is bounded above by the value 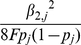. This can impose severe limitations on the power of any study in which there are such differences. To put this in perspective, consider two isolated populations which are each separated from their ancestral population with an F value of 0.0005, and consider an allele that exhibits 40 percent frequency in the ancestral population. The variance of the difference between the two isolated modern day populations in the frequency of this allele is equal to 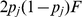 so that we would expect by chance that there is a about a 1.5 percent difference in allele frequencies between cases and controls for such an allele. Consider now the detection, in a study of 5,000 cases and 5,000 controls, of a disease-causing allele of the same frequency associated with a 10 percent difference in allele frequencies between cases and controls (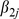 = .2). The difference in allele frequencies is approximately 6 times larger than expected due to population differences, and can be seen to correspond to an odds ratio for disease, under a multiplicative risk model, of 1.5 per copy of the risk allele. From equation (2) the non-centrality parameter 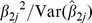 will be equal to 34.73 in this case; on the other hand if 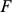 between cases and controls is 0 the non-centrality parameter will equal 208.33. Thus a study that would, given no differences between cases and controls in population of origin, have overwhelming power (>.9999) to reject the null hypothesis at a genome wide level significance (p<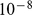) is, under this alternative, reduced to having power of only 56 percent after correcting for the differences in origins of cases and controls. The survey of European populations by Nelis et al [21] estimates fixation indices, 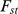, (which can be equated to 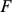 under the Balding Nichols model) between populations in SNP allele frequencies which range from less than .001 between neighboring populations to 0.023 for Southern Italy versus parts of Finland. Because our 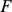 values as defined above are between present day and ancestral populations the fixation indices calculated between present day populations by Nelis et al need to be multiplied by ½ to be consistent with our definition of 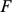. Thus the example we have given (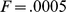) corresponds only to the nearest neighbor populations in Europe and would appear to throw into doubt any thought of using control data not perfectly matched in ancestry to cases.

### Admixed Populations

While the calculations given above appear to be pessimistic regarding the usefulness of shared control data it is important to note that the completely isolated population model is naïve and makes assumptions not applicable to the study subjects for the AABC study or indeed for most modern populations. Therefore we broaden our discussion to admixed populations, specifically, when the DNA from both cases and controls come from groups that are admixed from the same two ancestral populations. We consider this in two parts, first deriving results for comparisons between “completely” admixed populations, i.e. where the two populations have different levels of admixture between the ancestral populations, but when there is no within-population heterogeneity in ancestry. Next we focus on the much more realistic setting of incompletely admixed populations serving as cases and controls.

#### Completely admixed populations

Here we consider each of two populations of interest (one supplying cases and the other controls) as consisting of randomly mating populations derived by admixture from the same two ancestral populations. In the first modern day population we assume that 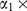
100 percent of the ancestors are from the first ancestral population and the remaining 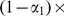
100 percent are from the other ancestral population. In the second population the fractions are 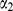 and 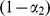 respectively. We further assume that the two ancestral populations are themselves derived from a single earlier ancestral population and that the 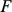 values of these populations compared to the earlier populations are both equal to the same value. Under these simplifying assumptions (which can be readily relaxed if need be) we can easily derive the covariance matrix for a SNP vector 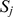 between subjects to have elements

Var(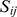) = 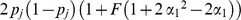 for subject 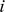 in the first population (i.e. cases)

Cov(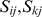) = 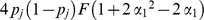 for both subjects 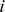 and 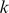 in the first population

Var(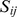) = 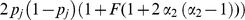 for subject 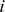 in the second population (i.e. controls)

Cov(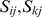) = 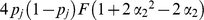 for both subjects 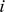 and 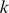 in the second population and finally

Cov(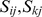) = 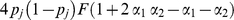 for subjects 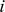 and 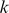 in different populations

With sampling of N cases from the first modern day admixed population and N controls from the second after repeated use of the matrix formulae for the inverse of a compound symmetric matrix we can compute the variance term for 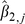 as the [2,2] element of the variance covariance matrix, 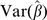,
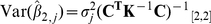
(6)as
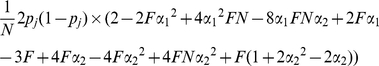
This expression is bounded from below by the quantity 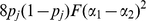.

We see that this bound is zero only if either 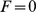 or if 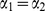. The non-centrality parameter will (as in the previous example) then again be bounded from above. Consider for example two groups of “completely” admixed modern-day subpopulations one with 25 percent of ancestry from one ancestral population (and 75 percent from the other population) and the other with 20 percent ancestry from the first ancestral population (and 80 percent from the second). If we take the fixation index, 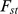 value of 0.153 of Nelis et al for (applicable for HapMap Yorubans versus HapMap Europeans) and the allele frequency and odds ratios as above (40 percent and 1.5 per copy respectively) then the non-centrality parameter 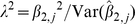 will be equal to 72.77 compared to 208.33 in the non-mixed case, leading again to loss of power although not as extreme as in the previous example.

#### Incompletely admixed groups

**Figure 1:**
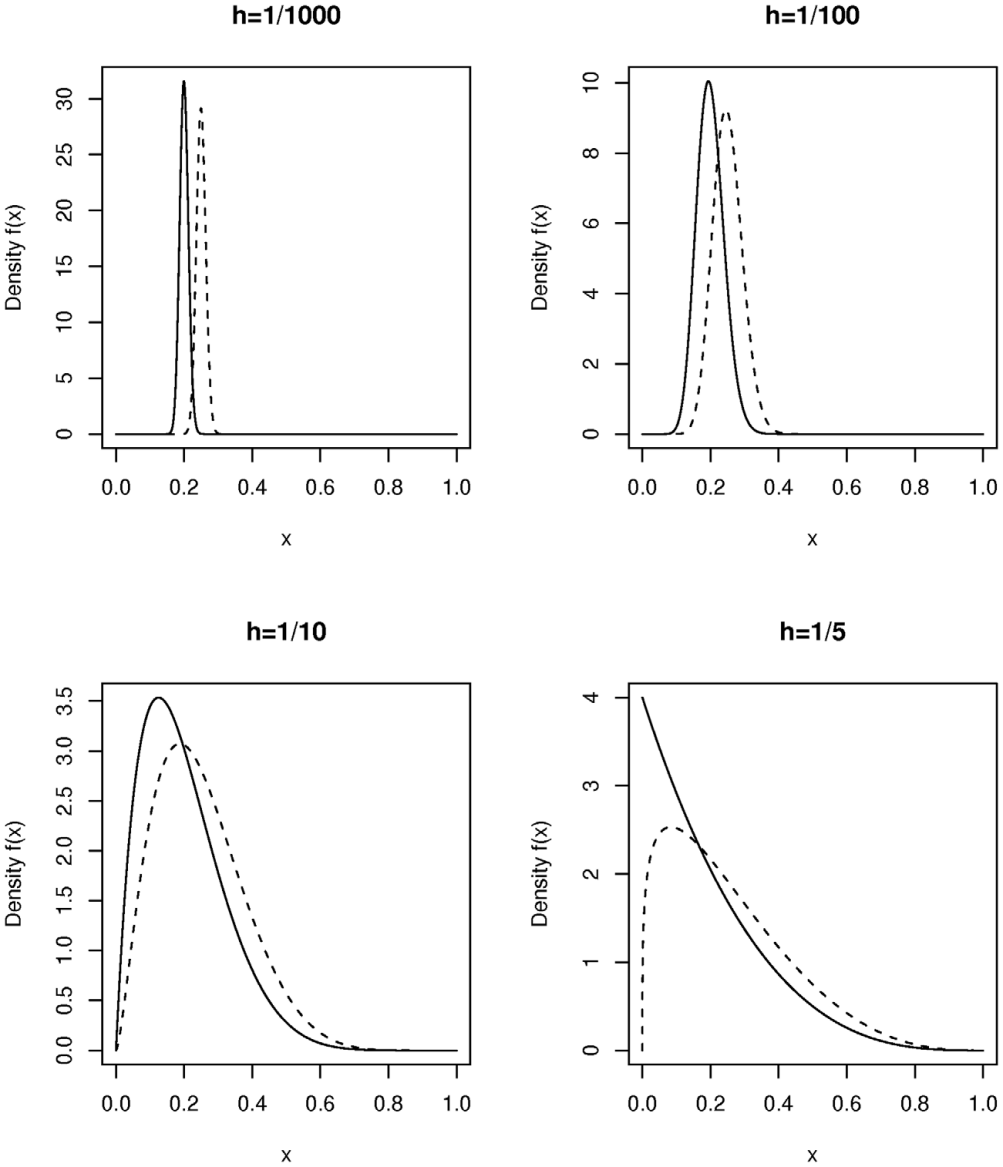
Plot of the density function of beta distributions parameterized by mean 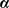 and heterogeneity factor 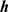. On each subplot the density is shown for two choices of 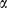 namely 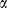 = 0.2 (solid line) and 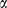 = 0.25 (dotted lines).

The above example is still unrealistic because we have assumed that all members within each of the two study populations (cases and controls) have exactly the same fraction of ancestry coming from the two differing ancestral groups: 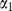 from population 1 for the cases and 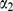 from population 1 for the controls. This is not what is seen in most complex population groups today, and is not what we see in the AABC data considered below. Instead in modern admixed populations there is a relatively wide range of within-population admixture proportions. If there is overlap due to ancestry for cases and controls then tests like the Bourgain test, principal components, multidimensional scaling [22], and genomic matching [23], [24] have a much greater likelihood of success than in the previous examples. Again we use the Bourgain test to quantify this. For example we can assume a distribution of values of the admixture fraction in the case and control samples while keeping a difference “on average” in ancestry between the two groups. We then can calculate “appropriate” values of the matrix K and use this in additional calculations. For our purposes the beta distribution provides a reasonably flexible model for the distribution of individual values of the admixture proportion in a modern day admixed population. Figure 1 gives a plot of the density function for a number of beta distributions. The beta distribution is usually described in terms of two parameters, 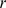 and 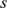 with the mean of the beta distribution equal to 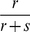 and the variance equal to 
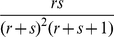
. For convenience we re-parameterize this distribution in terms of its mean, 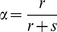, and a heterogeneity parameter, 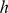, with 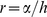, so that 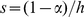. Thus samples from this distribution have mean 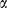 and variance 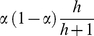. This parameterization is useful when comparing the overlap of two beta distributions with different means but similar within group variances as in Figure 1.

**Figure 2:**
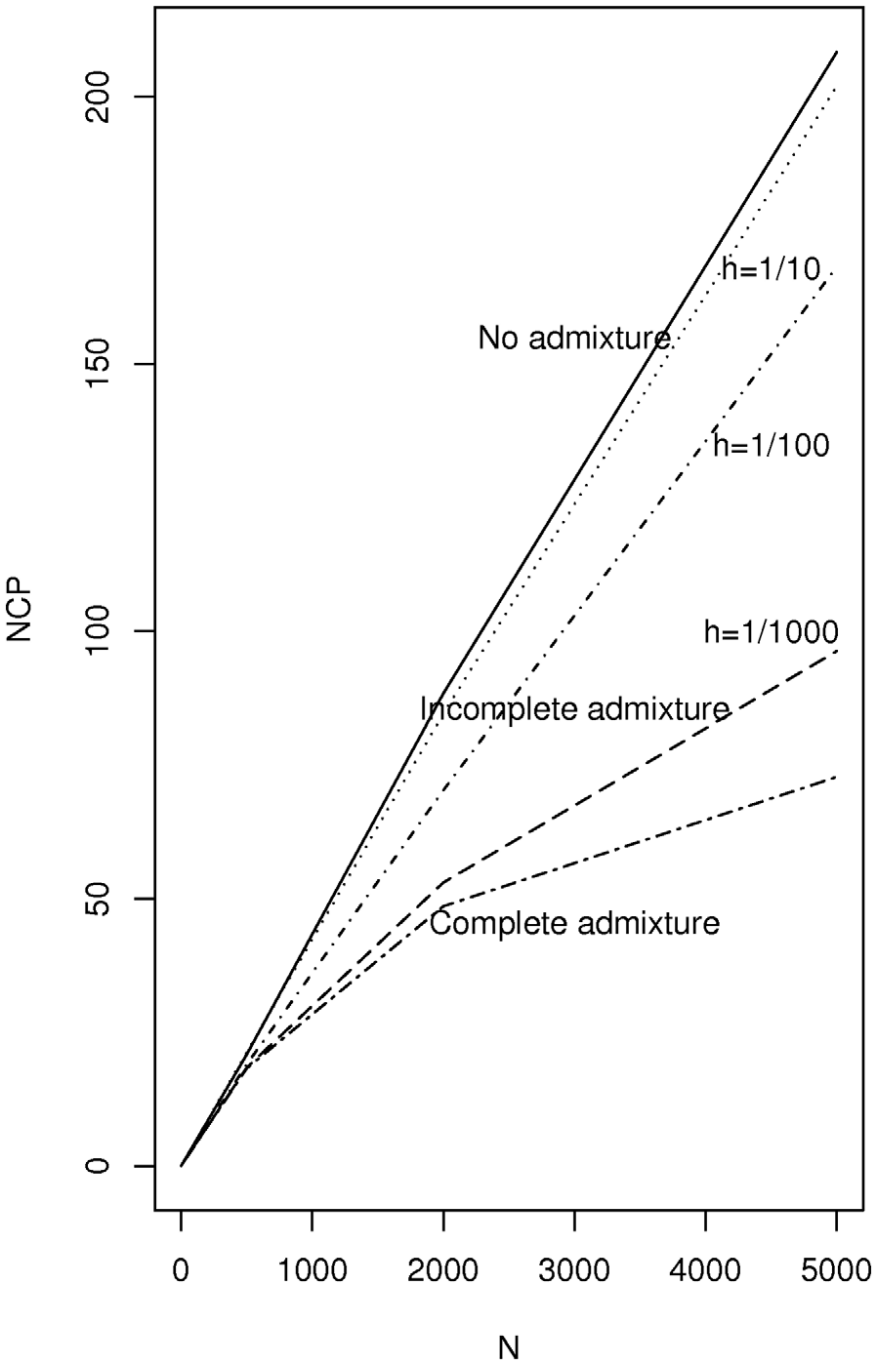
Plot of non-centrality parameter for the Bourgain test for a case-control study using two incompletely admixed populations as sources of cases and controls respectively. The parameters chosen refer to a test of a variant associated with disease which has 40 percent overall allele frequency and which is associated with a 10 percent difference in frequency between cases and controls (OR = 1.5 per copy). Cases are assumed to have average admixture percentage of 20 percent and controls 25 percent. The within population heterogeneity is specified by a single common heterogeneity parameter 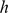 as used in Figure 1.

We consider now the non-centrality parameter for the Bourgain test when two incompletely admixed populations are used as cases and controls where there is an overall difference in admixture fraction in the two populations but where each population is heterogeneous with respect to the fraction of ancestry from the two mixing populations. Figure 2 plots the non-centrality parameter (NCP) for the Bourgain test against the number, 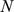, of cases and controls (1∶1 case-control ratio for 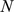 pairs) for the example used above (a risk variant of frequency 40 percent overall, and a OR for disease equal to 1.5 per copy) it is clear from the figure that the non-centrality parameter is very much determined by the degree of heterogeneity in admixture percentage within each population (cases versus controls) as parameterized here by 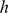.

### AABC Study Results

Our analysis focuses upon (1) estimating a more appropriate model for the distribution of ancestry in the data from the AABC data than the homogeneous “complete” admixture described above, (2) checking the adequacy of this model, enriching it if necessary, and (3) describing the implications of the model for the likely power to detect associations in studies in which all cases come from outside the AABC study populations, and controls are chosen from within the AABC. We can partly mimic such studies by making up “pseudo” case-control studies using the data from the different study sites within the AABC study.

#### Principal components analysis of the AABC study

**Figure 3:**
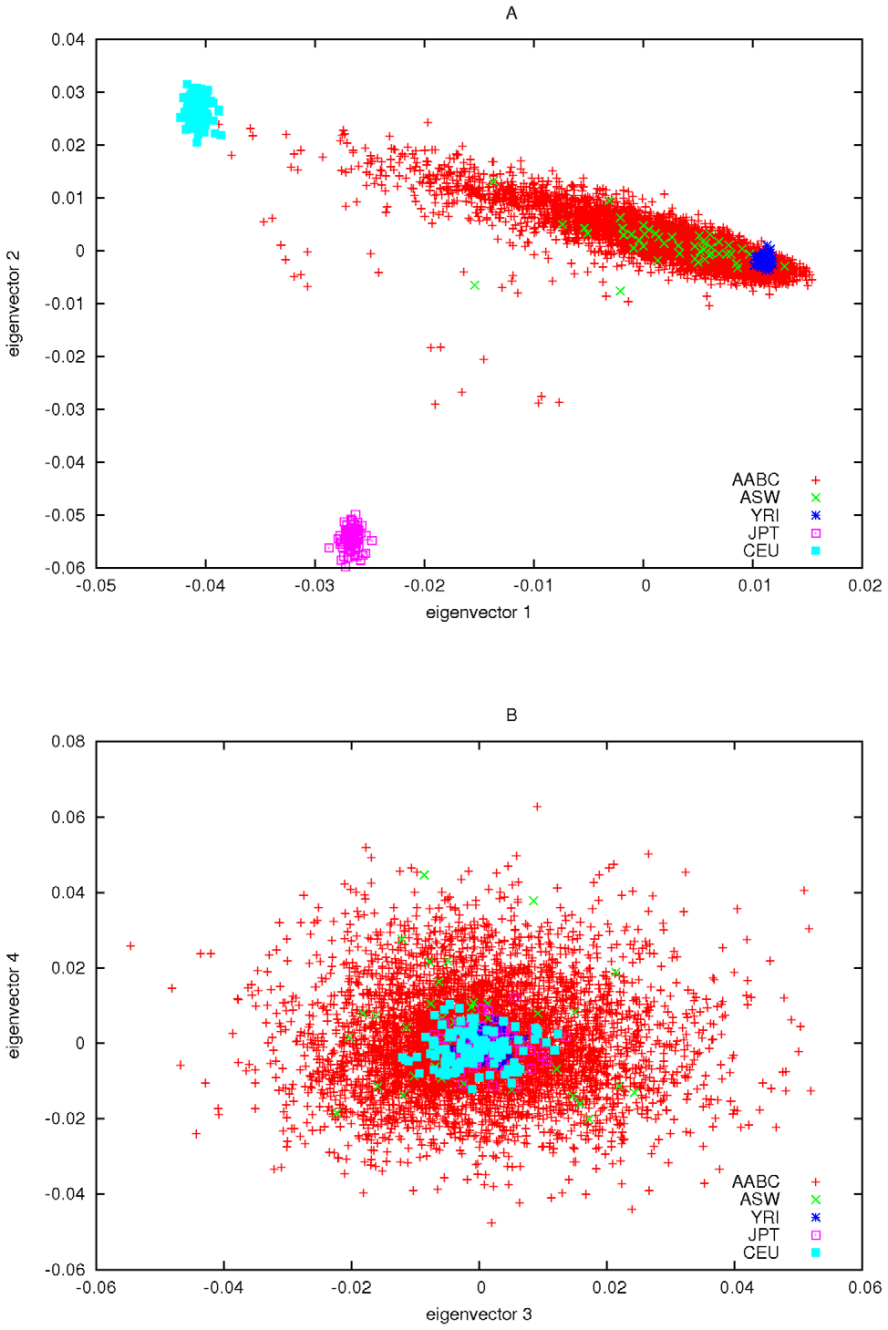
Principal components plots of AABC and selected HapMap samples.

We used the EIGENSTRAT program to calculate the eigenvectors and eigenvalues of the matrix 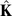 given in expression (3) using the set of 2,457 AIMs described above. We first noted that the first eigenvalue of 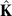 was much larger than the remaining eigenvalues (504.85 compared to 29.71 for the next largest). We strongly suspected that the eigenvector associated with this eigenvalue corresponds to the fraction of European ancestry based on CEU of HapMap. To clarify this we ran the program STRUCTURE on AABC study data and included HapMap genotype data (CEU, YRI, and CBT+YRI) for these same AIMs. The first eigenvector of the AABC data was highly correlated (r = 0.991) with the estimate of STRUCTURE for the percentage of European ancestry. For illustrative purposes we added the HapMap subject's data to the EIGENSTRAT analysis and plotted the first 4 eigenvectors in Figure 3.

#### Variation among AABC study sites in admixture percentage

**Figure 4:**
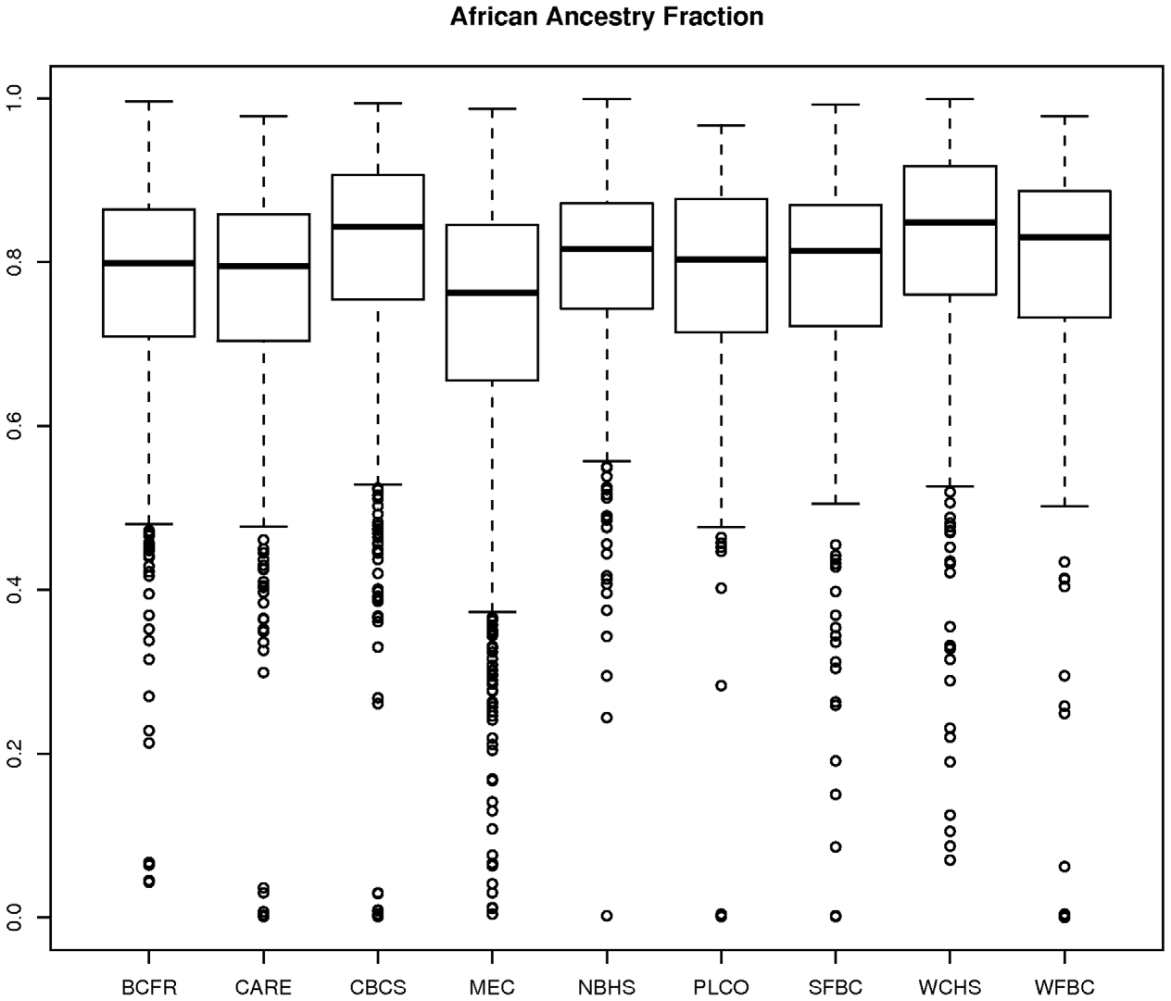
Plot of estimate of proportion of African ancestry from STRUCTURE by participating AABC study.

Figure 4 shows box plots of the STRUCTURE estimates of the fraction of European ancestry according to AABC study site. For each study site, we estimated the mean and variance of the distribution and related those by the method of moments to the two parameters (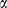 and 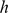) in our parameterization described above. The estimate 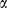 of average European ancestry varied significantly by study, from approximately 0.25 for the MEC cohort and other studies in California to approximately 0.19 for the CBCS and other studies in the East and South-east United States. The heterogeneity parameter also varied by site but was generally estimated to be close to 1/7 for all sites, which indicated a large degree of overlap between the admixture fractions even between the most different studies.

#### Other population structure in the AABC study

If the only ancestry differences among the control populations sampled by the AABC study relates to fraction of European versus African ancestry then our previous analysis would indicate that very little loss of power, compared to using perfectly matched controls, would result from a case-control study that uses African American cases, and one (or all) of the AABC control samples as convenience controls, after adjustment for differences in European versus African ancestry. Figure 3 gives evidence for some degree of admixture with an Asian as well as European group, which we assume [10] is largely due to admixture with Native American or Hispanic populations. The requirement of controlling for this additional admixture will also affect the power of using the AABC study as a source of shared controls, as would other types of admixture or population stratification. In order to quantify the total effect of the significant eigenvectors in the AABC data we did the following: Using the Tracy-Widom statistic as incorporated into EIGENSTRAT we estimated the number of “significant” eigenvalues (i.e. those significantly larger than the remaining eigenvalues) and hence the “significant” eigenvectors. Using these significant eigenvectors we formed a “smoothed” version of the matrix 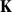 as
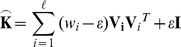
(7)where 
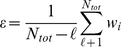
. Here 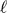 is the number of significant eigenvectors of 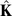, the 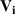, are the eigenvectors and 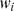 is the associated eigenvalue. Formula (7) gives a full rank estimate which has the same first 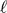 eigenvectors and eigenvalues as 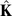 as well as the same trace (sum of all N eigenvalues). We use this to estimate non-centrality parameters for several example studies, based on the AABC data. We found that approximately the first 200 eigenvalues were nominally significant (at a 5 percent type I error rate) using the Tracy-Widom statistic and considered the effect of adjusting for a range of 1 to all 200 eigenvectors in our calculations. Fewer than all 200 eigenvectors likely to needed in a realistic analysis, here we are examining an extreme case. The fact that this many eigenvectors are nominally significant may be due in part to not controlling for multiple comparisons or may also be reflective of unsuspected close or distant relatedness between certain participants.

**Figure 5:**
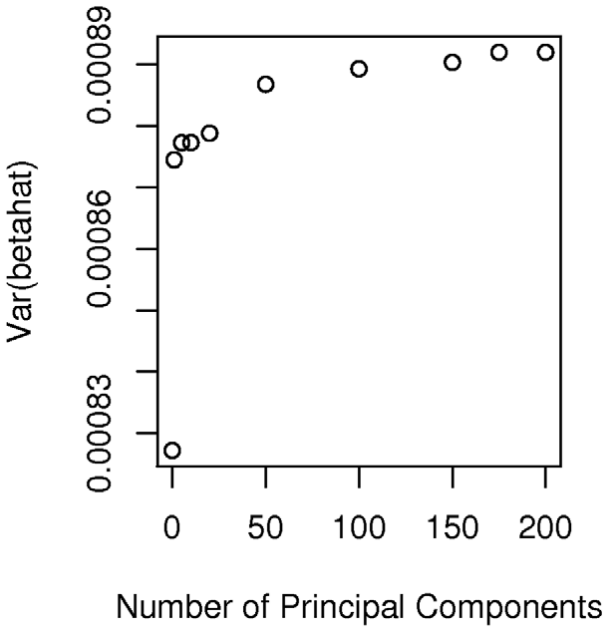
Plot of variance. Plot of the variance of 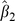 according to the number, 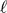
_,_ of eigenvectors adjusted for in the Bourgain test of association.

**Figure 6:**
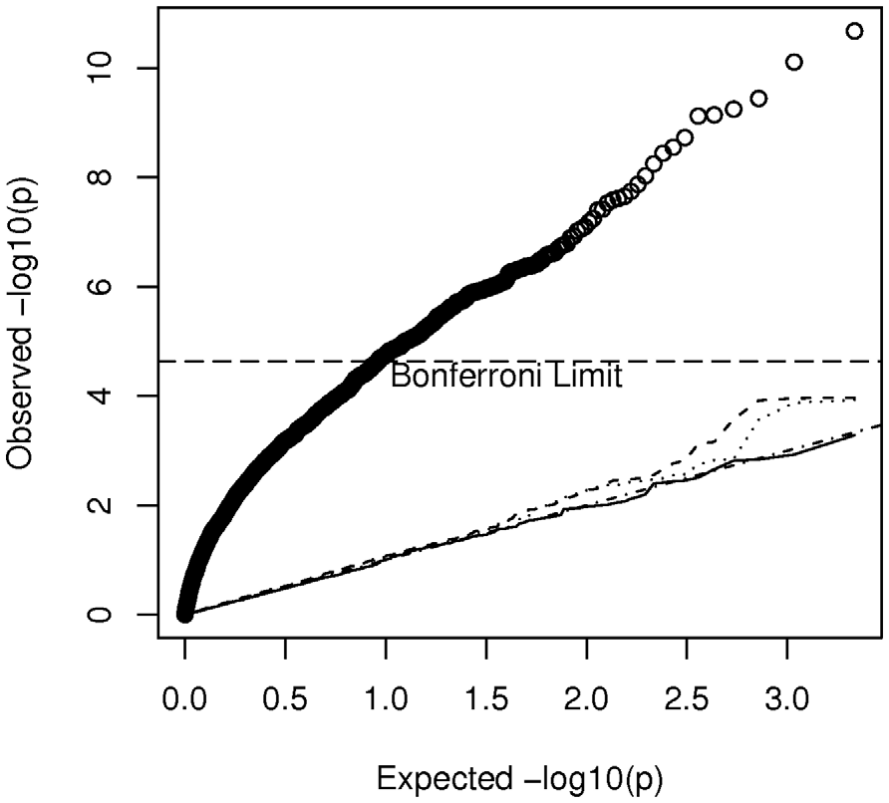
Quantile-quantile plot of p-values from association tests in the hypothesized case-control study in which cases from the CBCS and controls from the MEC are used. The plotted values indicate adjustment for 0 (uncorrected heavy solid line), 1 (dashed line), 10 (dotted line) and 200 (thin solid line) eigenvectors, by using these components in the calculation of 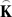.

Specifically we considered a breast cancer study in which all African-American cases (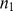 = 635) came from the CBCS and all controls (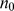 = 990) came from the Los Angeles component of the MEC. Accordingly we extracted the 1625 by 1625 submatrix (corresponding to these selected cases and controls) of 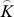 for use in the calculation of noncentrality parameters. The basic idea is to calculate 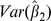 using the estimate of 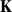 for this study, compared to a study of the same size but where cases and controls are sampled from the same non-admixed population. Because the appropriate 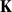 and 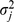 is not known, but is estimated from the data, our analysis of power becomes slightly more complicated than above. For a given SNP, 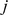, we estimated the variance of 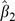 by the scalar
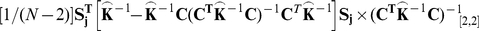
(8)The expected value of this variance estimate can be written as
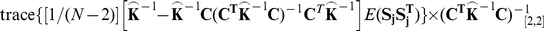
(9)The expected value 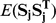 depends upon the details of admixture and other types of population structure in this subset of AABC participants and can be written as
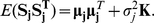
(10)In a study of homogeneous non-admixed case and control populations, and under the null hypothesis that 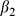 is zero, this reduces to 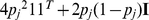 where 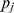 is the allele frequency of marker 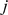. Substituting expression (10) into expression (9) with either 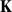 = 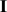 (for a homogeneous study) or for 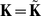 (for our example) allows us to compare the variance of the estimators when a total of 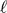 eigenvectors are adjusted for in our hypothesized case-control study, compared to the homogeneous study. Figure 5 plots the values of expression (9) for varying values of 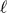 for a marker with frequency 20 percent. It appears that correcting for increasing numbers of eigenvectors (at least beyond the first two components) has relatively little effect on 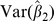 and hence on study effective sample size and power. Even using all 200 “significant” eigenvectors in the calculation of the smoothed estimator 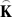 only increases the variance of the estimator by about 7 percent relative to a study that needs no correction. Of course we also need to confirm that the false positive rate for the Bourgain test is properly controlled for by using a particular number, 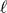, of eigenvectors. Figure 6 gives a quantile-quantile (QQ) plot of the (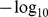) p-values from tests of association of each of the AIMs used to estimate K with case-control status in our hypothesized study, while using either 0 (uncorrected), 1 (the CEU – YRI component), 10, or 200 eigenvectors to form 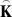. Here the uncorrected analysis is very highly over-dispersed with a very large number of associations globally significant (using the Bonferonni test). However even using just 1 eigenvector appears to give a reasonably adequate control of the type I error while correcting for 200 gives nearly perfect control, with little loss of additional power (as indicated in Figure 5).

## Discussion

We have adopted a somewhat non-standard approach in relying upon the Bourgain test rather than principal components [8] or related methods [22]–[24] to control for population structure in a GWAS of a minority population with cases/controls drawn from multiple studies with different designs and recruitment approaches. We have done this mainly because we can give certain theoretical results for the Bourgain test when assuming specific forms for the true kinship matrix 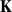 using this procedure. It is worth noting that the Bourgain test can be regarded as a random effects version of the usual principal components method. In particular the Bourgain model can be alternatively described as a model for the mean of 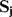 conditional upon all 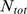 eigenvectors of 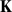 as
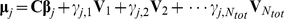
(10)Now consider the coefficients 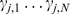 as being independent random effects with mean zero and variances equal to 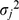 times the associated eigenvalues, 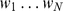 of 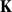. Averaging over all the random 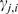 will yield the unconditional mean 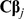 and variance covariance matrix 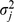 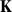. Our “smoothed” estimate, 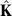, of 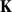 is motivated by expression (10), and choosing a value of 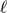 is analogous to choosing the number of eigenvectors to be used as fixed effects by EIGENSTRAT. The random effects framework also highlights the relationship between the Bourgain procedure and the genomic control method of Devlin and Roeder [25]. In genomic control one additional parameter that governs the dispersion of the test statistic is used to assess the association between 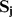 and case-control status. In the smoothed version of the Bourgain test introduced here, we choose a total of 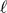 such parameters.

We have shown that if cases and controls come from genetically distinct populations but ones that have only recently diverged (so that the parameter 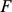 is very small) then some limited power remains to detect true marker associations so long as the true value of 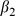 is very large compared to the “typical” differences between cases and controls seen with the other markers. This is also analogous in interpretation to the genomic control method. However our explicit description of the upper bound on the noncentrality parameter of the association test for such a study clearly shows the limits of this design, and by implication, the limits of the genomic control procedure as well. Basically neither of these two methods behaves “properly” from a statistical point of view as sample size increases, i.e. the non-centrality parameter under an alternative hypothesis (and hence power) does not increase correspondingly. In genomic control the overdispersion parameter that the procedure corrects for increases with sample size, while for the Bourgain test the noncentrality parameter is bounded from above.

For case-control studies involving two or more similarly admixed populations that differ in admixture fraction, the key issue in assessing the power of a study using cases from one population and controls from another is in determining the within-population heterogeneity of the admixture fractions, relative to the between population differences in average admixture. If the within-population heterogeneity is small then the situation is equivalent to the case of isolated populations, i.e., there will be a bound on the power of a study to detect an effect with the bound determined by the upper limit on the noncentrality parameter as a function of 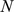 as computed above.

Despite the concerns raised in our theoretical considerations, in our assessment of the observed marker data from the AABC study we tentatively conclude that reuse of controls data from this study in future work may be statistically feasible. While there are clear differences in average admixture fraction between studies these are dwarfed by the within-study heterogeneity. Other signs of hidden structure in the AABC studies (as evidenced by additional eigenvalues which are significant by the Tracy-Widom test) do not appear to have a very large impact (Figure 5) on the power of our hypothetical study using the CBCS and MEC cases and controls respectively. Control for the first few (1–200 in our case) eigenvectors appears to dramatically reduce false positive associations with very little power loss (about a 7 percent reduction in effective sample size) relative to studies of homogeneous sets of cases and controls. We have used a specific set of ancestry informative markers in our analysis but the existence of genome-wide data for the AABC allows for considerable latitude in selecting SNPs to control for admixture, and even randomly selected SNPs, if enough are considered, can be used for admixture correction. Our use of the Bourgain test when considering the feasibility of a particular study design allows us to consider noncentrality parameters (and hence power) in particularly simple and helpful ways. While we have focused on the Bourgain method to correct for admixture differences in the AABC study our specific finding (that little loss in power is anticipated when re-using control data from this study) is likely to apply also to fixed-effects methods such as treating principal components or STRUCTURE estimates of percentage ancestry from ancestral populations as covariates. Our reasoning is based upon the close relationship between the principal components methods and the random effects rationale for the Bourgain test as given in equation (10) and also on the high correlation seen between STRUCTURE estimates of African ancestry in the AABC study and the first eigenvector from principal components.
